# Calibration and Evaluation of Quantitative Antibody Titers for Varicella-Zoster Virus by Use of the BioPlex 2200

**DOI:** 10.1128/JCM.00296-19

**Published:** 2019-07-26

**Authors:** Elizabeth McLachlan, Heidi Scholz, Shelly Bolotin, Natasha S. Crowcroft, Todd F. Hatchette, Colleen Jackson, Alberto Severini

**Affiliations:** a National Microbiology Laboratory, Public Health Agency of Canada, Winnipeg, Manitoba, Canada; b Bio-Rad Laboratories, Benicia, California, USA; c Public Health Ontario, Toronto, Ontario, Canada; d Dalla Lana School of Public Health, University of Toronto, Toronto, Ontario, Canada; e Department of Laboratory Medicine and Pathobiology, University of Toronto, Toronto, Ontario, Canada; f Institute for Clinical Evaluative Sciences, Toronto, Ontario, Canada; g Department of Pathology, Dalhousie University, Halifax, Nova Scotia, Canada; h Department of Pathology and Laboratory Medicine, Nova Scotia Health Authority, Halifax, Nova Scotia, Canada; i Department of Medical Microbiology, University of Manitoba, Winnipeg, Manitoba, Canada; Memorial Sloan Kettering Cancer Center

**Keywords:** varicella-zoster virus, BioPlex, quantitative, antibody titer, immunity, seroepidemiology

## Abstract

Most commercially available enzyme immunoassay-based methods have limited sensitivity to detect antibody responses to varicella-zoster virus (VZV) in vaccinated individuals, who produce lower antibody levels than those with natural infection. However, more sensitive methods are either not commercially available or less amenable to high-throughput testing. The BioPlex 2200 measles, mumps, rubella, and varicella (MMRV) IgG assay (Bio-Rad Laboratories, Hercules, CA) is an automated high-throughput platform based on the microsphere Luminex technology that measures antibodies against measles, mumps, rubella, and varicella viruses simultaneously. Although it has U.S. Food and Drug Administration approval as a qualitative diagnostic test for measles, mumps, rubella, and varicella virus immunity, in this study, we have validated the assay to produce quantitative titers (off label) against the VaccZyme VZV glycoprotein (VZVgp) low-level IgG kit (The Binding Site Ltd., Birmingham, UK) using the World Health Organization international standard. Here, we show that the BioPlex 2200 MMRV IgG assay has sensitivity superior to that of the Zeus enzyme-linked immunosorbent assay (ELISA) VZV IgG assay (Zeus Diagnostics, Branchburg, NJ). Using receiver operating characteristic (ROC) analysis and adjusting the cutoff levels, we improved the sensitivity of the quantitative BioPlex 2200 MMRV IgG assay to 97.4%, while maintaining 100% specificity.

## Abstract (precis)

Most commercially available enzyme immunoassay-based methods have limited sensitivity to detect antibody responses to varicella-zoster virus (VZV) in vaccinated individuals, who produce lower antibody levels than those with natural infection. However, more sensitive methods are either not commercially available or less amenable to high-throughput testing.

## INTRODUCTION

Detection of IgG antibodies against varicella-zoster virus (VZV) is routinely performed to determine immunity status in occupational screening (e.g., health care workers) and the risk of infection in vulnerable patients (e.g., transplant patients and pregnant women), and it can also be used to determine the immunity status of a population in seroepidemiological studies (1).

The importance of determining immune status has increased since 1998, when a live attenuated VZV vaccine, developed in 1974 (2) and first licensed in 1984 (3), started to be routinely administered in Canada (4), either alone or in combination with the measles, mumps, rubella, and varicella (MMRV) vaccine. In countries that have implemented universal 2-dose VZV vaccination, varicella incidence has declined by about 90%, and secondary vaccine failure causes almost exclusively mild varicella cases (5, 6).

Vaccine-induced immunity produces VZV IgG titers that are lower than those for the natural disease (7, 8), and, due to this, commercial enzyme immunoassay (EIA)-based assays for VZV IgG may not be sensitive enough to detect immunity in a percentage of vaccinated individuals, possibly leading to an underestimation of immunity in highly vaccinated populations (8, 9). More sensitive gold standard assays, such as fluorescent-antibody-to-membrane-antigen (FAMA) (10, 11) and time-resolved fluorescence immunoassay (TRFIA) (12, 13), have been used to determine the immune status in vaccinated individuals with greater sensitivity. However, these gold standard tests are not all commercially available and are not suitable for high-throughput testing, as they are labor-intensive.

A glycoprotein EIA (gpEIA) developed by Merck uses purified VZV glycoproteins from VZV-infected cells to reliably detect the protective IgG response elicited by the VZV vaccine (14–16). This assay is not available to most laboratories, but a kit based on the same principle is available commercially as the VaccZyme VZVgp low-level IgG kit (The Binding Site Ltd., Birmingham, UK). This kit has been shown to have a sensitivity equivalent to those of FAMA and TRIFA for the detection of VZV IgG in serum samples from people with previous natural infection, but it was somewhat less sensitive in detecting immunity in vaccinated people if the manufacturer’s positivity threshold was used (12, 17). However, the VaccZyme gpEIA titers were shown to correlate strongly with FAMA results, indicating that lowering of the positivity threshold would also detect most of the low-titer immune individuals (18).

The BioPlex 2200 system (Bio-Rad Laboratories, Hercules, CA) is an automated high-throughput platform based on the microsphere Luminex technology, allowing the determination of multiple analytes in a single reaction. The BioPlex 2200 MMRV IgG assay measures antibodies against measles, mumps, rubella, and varicella virus simultaneously. This test compares favorably to other commercial EIA kits, and it has been approved by the U.S. Food and Drug Administration as a diagnostic test for MMRV immunity as a qualitative method returning positive, negative, or equivocal results (19, 20). However, quantitative determination of continuous IgG titers would be more suitable for seroepidemiological studies so that mathematical models can be applied to calculate the susceptibility thresholds of a population. The susceptibility threshold can also be calculated for individual cohorts within a population, such as by age group (21–23).

We have previously shown that anti-measles IgG results for the BioPlex 2200 MMRV IgG assay can be converted from relative fluorescence intensity (RFI) units into quantitative data that correlate well with the degree of measles virus immunity, although reference testing with plaque reduction neutralization assay is required to resolve equivocal BioPlex measles virus IgG results (24).

In this study, we calibrated the BioPlex 2200 MMRV IgG assay to provide a quantitative measurement of VZV IgG antibodies, and we compared it with the commercial VaccZyme gpEIA method and with the Zeus enzyme-linked immunosorbent assay (ELISA) varicella-zoster IgG test system (Zeus Diagnostics, Branchburg, NJ), which is commonly used for VZV serology testing in clinical laboratories.

## MATERIALS AND METHODS

### Study design.

An initial validation set of 148 anonymized residual serum samples submitted for routine testing of VZV immune status and previously categorized as immune, nonimmune, or equivocal using the Zeus Diagnostics varicella-zoster virus IgG EIA (Zeus Diagnostics, Branchburg, NJ) were tested by the BioPlex 2200 MMRV IgG assay at the Nova Scotia Health Authority QEII Microbiology Laboratory (Halifax, Nova Scotia, Canada) and by VZV gpEIA (VaccZyme, Binding Site, Birmingham, UK) at the National Microbiology Laboratory (NML) in Winnipeg, Manitoba, Canada. The local institutional review board provided ethics approval for the use of anonymized residual serum samples for this study. A second set of 1,199 anonymized residual specimens collected for the purposes of a seroepidemiology study from the Province of Ontario, Canada, were tested by the BioPlex 2200 MMRV IgG assay at the NML. Specimens below the positivity cutoff of 190 mIU/ml established with the initial validation set (see Results) were retested using VaccZyme gpEIA. The use of these specimens was approved by the local research ethics boards at the University of Toronto and the University of Manitoba.

### BioPlex 2200 MMRV IgG kit.

The BioPlex 2200 MMRV IgG assay is a multiplex flow immunoassay that simultaneously detects and identifies antibodies against measles, mumps, rubella, and varicella viruses in a single test reaction (19, 20). The BioPlex 2200 system combines 5 μl of patient sample with sample diluent and a reagent containing a population of four different dyed microspheres coated with different antigens to detect the presence of IgG antibodies for measles, mumps, rubella, and varicella-zoster viruses. The dyed bead identity is determined by the fluorescence of the dyes, and the quantity of antibody captured by the antigen is determined by the fluorescence of an anti-human IgG-phycoerythrin-labeled conjugate. Raw data are calculated in relative fluorescence intensity (RFI). When run on the BioPlex 2200 instrument, the RFI is normalized to an antibody index (AI), which is a qualitative numeric result, using a two-level calibration curve. The AI values are displayed to the operator. The sample AI result is compared to negative and positive ranges established by the manufacturer, <0.9 AI (negative) and ≥1.1 (positive), to generate a qualitative status (positive, negative, or equivocal). The generation of the calibration curve is necessary to standardize RFI and correct for variation between runs and reagents. For the purpose of this study, we used the RFI values from the BioPlex 2200 MMRV IgG test results to generate a calibration curve using dilutions of the WHO VZV IgG international standard (25), which allowed us to calculate quantitative antibody titers in milli-international units per milliliter.

### VaccZyme VZVgp low-level IgG EIA.

The VaccZyme VZVgp low-level IgG enzyme immunoassay kit, produced by The Binding Site Group Ltd., Birmingham, UK, uses affinity-purified glycoproteins from VZV-infected cell lines as an antigen (15, 26). Because glycoproteins are the main antigens for VZV-neutralizing antibody, a gpEIA maximizes sensitivity for detecting the VZV IgG immune response. The VaccZyme gpEIA is designed to detect low levels of VZV IgG antibodies, and it is quantitative between 10 and 810 mIU/ml, based on the first WHO VZV international standard (25). The protective level of antibodies was set by the manufacturer at ≥150 mIU/ml, and the susceptibility threshold was set at <100 mIU/ml, based on the agreement with the TRFIA VZV assay (12, 17). The test was used according to the instructions of the manufacturer. Briefly, serum samples are diluted 100× and added to wells coated with VZV glycoprotein antigen. Unbound antibody is washed off after 30 min, and peroxidase-conjugated anti-human IgG is added. The color reaction from the addition of substrate is measured at 450 nm, and the optical density is calibrated against a standard curve to give quantitative results in the range of 10 to 810 mIU/ml.

### Statistical analysis.

Regression lines were fitted using Excel (Microsoft, Inc.) or the Prism 7 (GraphPad Software, Inc.) software. ROC analysis was performed using Prism 7.

## RESULTS

### Comparison between BioPlex 2200 VZV IgG and VaccZyme gpEIA.

The BioPlex 2200 test for VZV IgG was developed as a qualitative method to detect VZV immune serum based on the measurement of IgG fluorescence using an arbitrary antibody index (AI) (19, 20).

**FIG 1:**
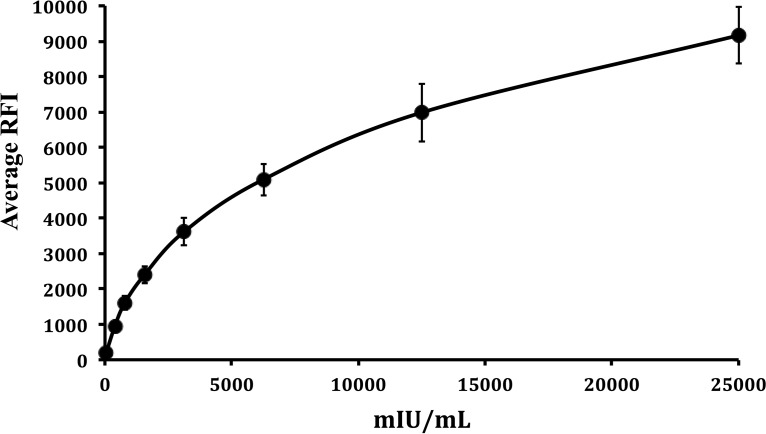
Standard reference curve for the quantitative BioPlex 2200 VZV IgG assay. Relative fluorescence intensity measured for 2-fold serial dilutions of the first WHO VZV IgG international standard. Each point was measured in triplicate, and the average ± standard deviation (SD) values are shown. The curve was fitted using a 4-parameter logic log equation.

**FIG 2:**
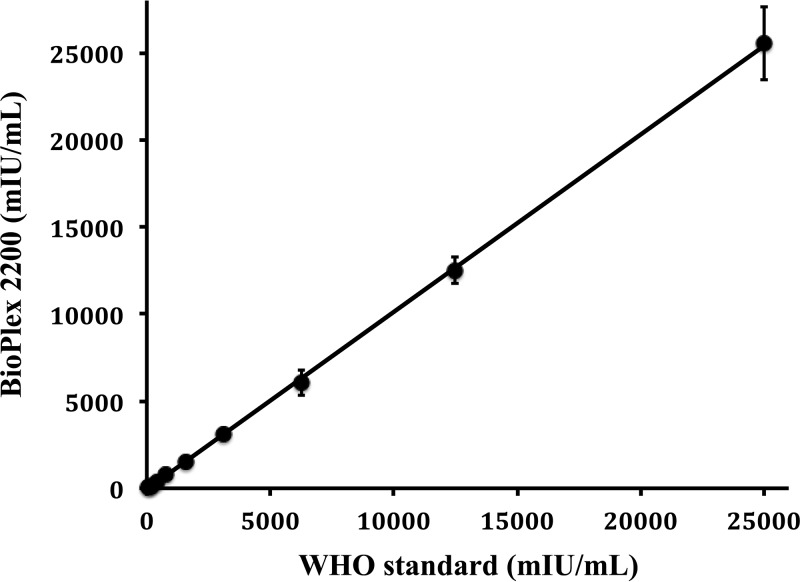
Linearity of the quantitative BioPlex 2200 VZV IgG test. Two-fold dilutions of the first VZV international standard were run on the BioPlex 2200, and the RFI were transformed into milli-international units per milliliter using the calibration curve described in Fig. 1. The results show an almost perfect linear correlation coefficient and a slope near 1.00, indicating that the BioPlex method is linear at least up to 25,000 mIU/ml.

To develop a BioPlex 2200 quantitative test for VZV antibodies, we first generated the calibration curve shown in Fig. 1 by testing a 2-fold serial dilution (from 0.05 IU/ml to 25 IU/ml) of the World Health Organization (WHO) international standard for VZV IgG (25). The best fit of the calibration curve (4-parameter logic log) was used to transform RFI units, as measured by the BioPlex 2200 into milli-international units per milliliter of VZV IgG. To verify the accuracy of the calibration curve, the WHO VZV international standard serial dilutions were retested by BioPlex 2200 in triplicate and transformed to milli-international units per milliliter. Figure 2 shows an almost-exact correlation between measured and expected milli-international units per milliliter levels (*R*^2^ = 0.9998, slope = 1.018).

**FIG 3:**
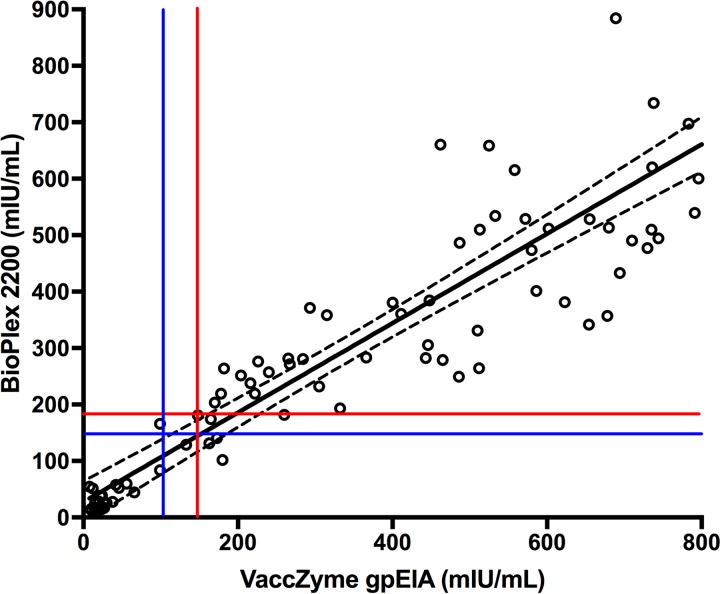
Comparison between the BioPlex 2200 and the VaccZyme gpEIA, using a validation set of 148 archival specimens. The vertical blue and red lines identify the VaccZyme gpEIA equivocal zone suggested by the manufacturer, between 100 and 150 mIU/ml, respectively. The horizontal red line at 190 mIU/ml marks the positive cutoff that defines a positive agreement with gpEIA of 97.4%. The horizontal blue line marks the negative cutoff of 152 mIU/ml, which identifies a negative agreement of 100% with the VaccZyme gpEIA (see also Table 1). The dashed lines represent the 95% CI of the regression line.

We then compared the performance of the BioPlex 2200 to that of the VaccZyme gpEIA using a panel of 148 residual serum samples that were previously classified as nonimmune (*n* = 50), equivocal (*n* = 50), or immune (*n* = 48) by the qualitative commercial kit Zeus ELISA varicella-zoster IgG, according to the manufacturer’s instructions. These serum samples were tested in parallel by the VaccZyme gpEIA and by the BioPlex 2200 VZV IgG, and the BioPlex 2200 readings were transformed into milli-international units per milliliter using the calibration curve shown in Fig. 1. The results are shown in Fig. 3. There is a linear correlation between the BioPlex 2200 and VaccZyme gpEIA (*R*^2^ = 0.787, *P* < 0.0001), with a slope of 0.792 (95% confidence interval [95% CI], 0.784 to 0.88), significantly lower than 1, as expected given the reported higher sensitivity of gpEIA methods in comparison with whole-VZV-antigen methods, such as the BioPlex 2200 assay (15).

**FIG 4:**
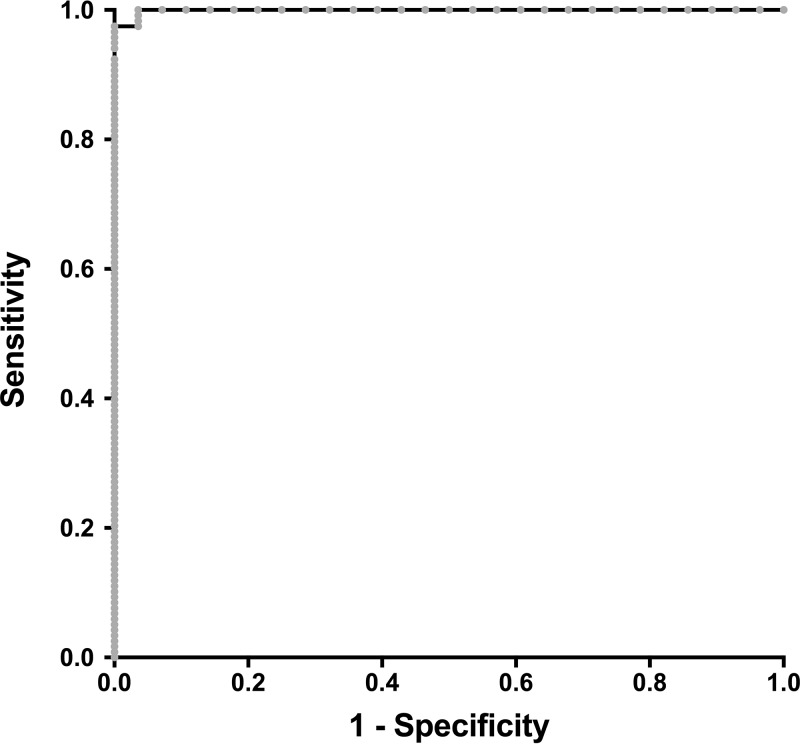
ROC analysis of the BioPlex 2200 results against the VaccZyme gpEIA results. The area under the curve is 0.999. Results were classified as positive or negative for VZV protective immunity using the manufacturer’s cutoff for the VaccZyme VZV gpEIA of 150 mIU/ml and 100 mIU/ml, respectively. Two samples in the equivocal range were excluded from the ROC analysis.

The adequate correlation between the VaccZyme gpEIA and BioPlex 2200 results is confirmed by a receiver operating characteristic (ROC) analysis in which serum samples with a gpEIA titer equal to or above 150 mIU/ml were considered immune, and serum samples with a gpEIA titer below 100 mIU/ml were considered nonimmune, according to the instructions of the manufacturer. Two gpEIA equivocal results were excluded from the analysis. The ROC curve, displayed in Fig. 4, shows an excellent correlation, with an area under the curve of 0.999 (95% CI, 0.997 to 1.001). By setting the BioPlex 2200 positivity cutoff at 190 mIU/ml (Fig. 3, red line) and the negativity cutoff at 152 mIU/ml (Fig. 3, blue line), we obtained optimum positive and negative agreements with the VaccZyme gpEIA of 100% and 97.4%, respectively, with only 2 specimens falling in the equivocal range (Table 1).

**TABLE 1:** Agreement between quantitative BioPlex 2200 VZV IgG assay and VaccZyme gpEIA results

VaccZyme gpEIA result	BioPlex 2200 VZV IgG result (no.)^*a*^			
	Positive	Equivocal	Negative	Total
Positive	113	2	3	118
Equivocal	0	1	1	2
Negative	0	1	27	28
Total	113	4	31	148

a Positive agreement at the BioPlex cutoff of 190 mIU/ml, 97.4% (95% CI, 92.7 to 99.1%). Negative agreement at the BioPlex cutoff of 152 mIU/ml, 100% (95% CI, 87.7 to 100%). Equivocal results were considered negative for the calculation of positive agreement and positive for the calculation of and negative agreement.

Our laboratory-developed quantitative cutoff values correspond to BioPlex 2200 antibody index (AI) values of 0.7 and 0.6, respectively, which are lower than the AI values of 1.1 and 0.9 recommended by the manufacturer for the qualitative VZV IgG assay. Tables 2 and 3 show how the positive agreement between BioPlex 2200 and VaccZyme gpEIA increases from 76.3% (95% CI, 67.4 to 83.4%) to 94.1% (95% CI, 87.7 to 97.3%) by lowering the qualitative threshold of positivity to an AI of 0.7, while the negative agreement remains unchanged at 100%. The positive agreement between the VaccZyme gpEIA and the Zeus diagnostic kit was 40.8% (95% CI, 31.8 to 50.1%), which shows the greater sensitivity of the BioPlex 2200 (Table 4). Thus, by lowering the threshold of positivity of the qualitative assay, we were able to increase the sensitivity of BioPlex 2200 and still maintain 100% specificity in comparison to the gold standard gpEIA.

**TABLE 2:** Agreement between qualitative BioPlex 2200 VZV IgG assay and VaccZyme gpEIA, using the manufacturer's AI cutoff

VaccZyme gpEIA result	BioPlex 2200 VZV IgG result (no.)^*a*^			
	Positive (>1.1 AI)	Equivocal	Negative (<0.9 AI)	Total
Positive	90	13	15	118
Equivocal	0	0	2	2
Negative	0	0	28	28
Total	90	13	45	148

a Positive agreement with the BioPlex, 76.3% (95% CI, 67.4 to 83.4%). Negative agreement with the BioPlex, 100% (95% CI, 85.9 to 100%). Equivocal results were considered negative for the calculation of positive agreement and positive for the calculation of and negative agreement. AI, antibody index.

**TABLE 3:** Agreement between qualitative BioPlex 2200 VZV IgG assay and VaccZyme gpEIA, using lower AI cutoffs

VaccZyme gpEIA result	BioPlex 2200 VZV IgG result (no.)^*a*^			
	Positive (>0.7 AI)	Equivocal	Negative (<0.6 AI)	Total
Positive	111	4	3	118
Equivocal	0	1	1	2
Negative	0	1	27	28
Total	111	6	31	148

a Positive agreement with the BioPlex, 94.1% (95% CI, 87.7 to 97.3%). Negative agreement with the BioPlex, 100% (95% CI, 85.9 to 100%). Equivocal results were considered negative for the calculation of positive agreement and positive for the calculation of and negative agreement. AI, antibody index.

**TABLE 4:** Agreement between Zeus VZV IgG ELISA and VaccZyme gpEIA

VaccZyme gpEIA result	Zeus VZV IgG ELISA result (no.)^*a*^			
	Positive	Equivocal	Negative	Total
Positive	48	46	24	118
Equivocal	0	0	2	2
Negative	0	4	24	28
Total	48	50	50	148

a Positive agreement with the Zeus, 40.8% (95% CI, 31.8 to 50.1%). Negative agreement with the Zeus, 100% (95% CI, 85.9 to 100%). Equivocal results were considered negative for the calculation of positive agreement and positive for the calculation of and negative agreement. AI, antibody index.

**FIG 5:**
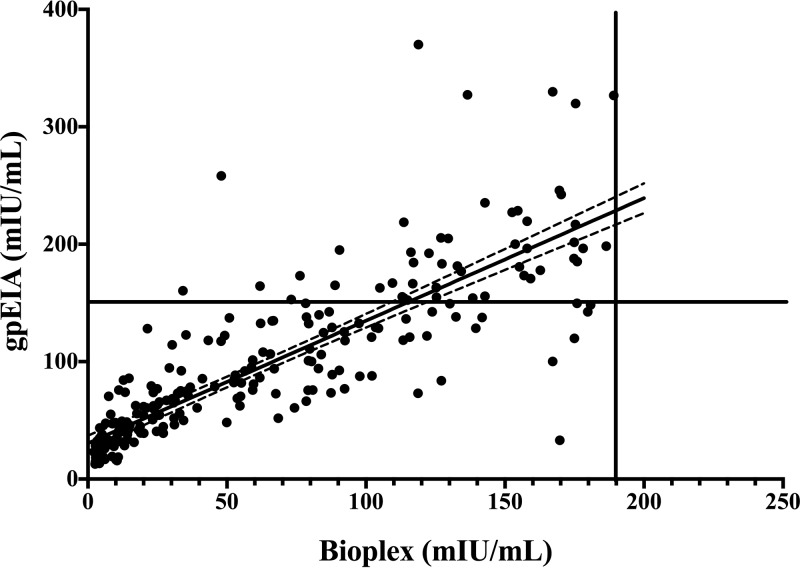
Correlation between BioPlex 2200 and VaccZyme gpEIA for low-titer samples. Samples that tested below the established cutoff of positivity for the BioPlex 2200 (Fig. 3 and Table 1) were retested by the VaccZyme gpEIA. The results show a significant linear correlation (*R*^2^ = 0.787, *P* < 0.0001). The vertical line marks the positive cutoff for the BioPlex 2200 (190 mIU/ml), and the horizontal line marks the positivity cutoff for the VaccZyme VZV gpEIA, at 150 mIU/ml.

To assess the performance of the quantitative BioPlex 2200 assay and VaccZyme gpEIA on samples with a range of immunity levels, including samples from unvaccinated, vaccinated, and naturally infected individuals, we performed a serosurvey using 1,199 residual serum samples obtained from Ontario, Canada. Of these, 259 specimens tested below the positivity threshold of 190 mIU/ml by the quantitative BioPlex 2200 assay. We retested these specimens using VaccZyme gpEIA, and the results are shown in Fig. 5. Forty-eight specimens tested positive by the VaccZyme gpEIA (i.e., above the horizontal line in Fig. 5), and 211 specimens remained negative by both methods. Assuming, as calculated from Table 1 and by the ROC curve, that all the BioPlex 2200 positive specimens are also gpEIA positive (*n* = 940), we calculated the positive agreement of BioPlex 2200 relative to the VaccZyme gpEIA to be 95.1% (95% CI, 93.6 to 96.4%) (Table 5), which is not significantly different from the positive agreement calculated with the validation panel shown in Table 1, 97.4% (95% CI, 92.7 to 99.1%). The linear correlation coefficients and the slopes of the linear regression in Fig. 3 and 5 are also very similar, confirming that the BioPlex 2200 VZV IgG performed as expected on a larger sample of specimens, based on the results obtained with the validation panel (Table 1 and Fig. 3).

**TABLE 5:** Comparison between quantitative BioPlex 2200 VZV IgG assay and VaccZyme gpEIA using a sample of low-titer specimens

VaccZyme gpEIA	BioPlex 2200 VZV IgG		
	Negative	Positive	Total
Negative	211	0^*a*^	211
Positive	48	940^*a*^	988
Totals	259	940	1,199

a Based on the validation shown in Fig. 2, BioPlex 2200 VZV IgG-positive specimens were assumed to be positive also by the VaccZyme gpEIA and were not retested.

## DISCUSSION

Serosurveys of VZV IgG antibodies are conducted to determine the level of immunity of a population. They are instrumental in informing vaccine policy, whether for assessing immunity in the general population (1, 27, 28), subpopulations, e.g., recent immigrants from tropical climates (29, 30), or immunosuppressed children who may benefit from VZV vaccination (30).

An ideal test for VZV serosurveys should be sensitive enough to detect antibodies produced after vaccination, which are of lower titer than antibodies produced by natural infection; it should allow for high throughput to handle large studies, and it should be quantitative to allow analyses of threshold of immunity and the levels of herd immunity.

The BioPlex 2200 MMRV IgG test meets the criteria of automation and high throughput. Our results with a validation set (Fig. 3 and Table 1) demonstrate that the BioPlex 2200 can measure VZV IgG in a quantitative manner when calibrated using a serial dilution of the VZV IgG international standard (Fig. 1 and 2). Quantitation by the BioPlex 2200 correlates very well with the sensitive VaccZyme gpEIA method. On the basis of the results with a validation set, we have determined the optimal cutoffs (152 mIU/ml for a negative result and 190 mIU/ml for a positive result) for the quantitative BioPlex 2200 method, which provided 97.4% positive agreement and 100% negative agreement with the VaccZyme gpEIA. The positivity threshold of 190 mIU/ml for quantitative use of the BioPlex 2200 VZV IgG test corresponds to a 0.7 AI threshold for the qualitative test, lower than the 1.1 AI normally used for the FDA-approved BioPlex 2200 VZV IgG diagnostic test. Testing by the BioPlex 2200 of a set of residual serum specimens and retesting by the VaccZyme gpEIA confirm the positive and negative agreements observed with the validation set (Table 5) and confirm the linear relationship between the VZV IgG titers measured by the BioPlex 2200 and VaccZyme gpEIA (Fig. 5).

In summary, our data show that the BioPlex 2200 can quantitatively measure VZV IgG titers with a sensitivity and specificity comparable to those of the VaccZyme gpEIA, provided the appropriate cutoffs are chosen. Lowering the positivity cutoff may increase the risk of overestimating individual immunity. Additional studies on vaccinated populations are needed to establish if the lower BioPlex 2200 positivity threshold is warranted. We have previously validated the BioPlex 2200 as a quantitative method for measuring measles virus IgG, using plaque reduction neutralization (PRN) as a reference test (24). However, there was poor correlation between the neutralization titers measured by PRN and the total IgG titer measured by the BioPlex 2200 for samples near the threshold of immunity, as is the case for other measles virus IgG EIA (31, 32). Therefore, reference testing by PRN is advisable to obtain a clear measurement of the percentage of serum samples that have protective titers of measles virus IgG. In the case of VZV antibodies, because of the good correlation and positive/negative agreement of the BioPlex 2200 with the reference test gpEIA, reference testing of negative and equivocal specimens does not increase the specificity or precision, suggesting that this more labor-intensive method is not necessary.

The BioPlex 2200 platform is best suited for use in large seroepidemiological studies where high throughput is required. Although the BioPlex 2200 is a specialized instrument and may be cost-prohibitive to some labs that do not currently have the instrument, the highly automated technology has significantly less hands-on time for processing samples; as such, the labor cost required to carry out the BioPlex assays is a fraction of the labor costs of the gold standard assays. The reagent costs per sample of the individual BioPlex MMRV tests and the VaccZyme gpEIA or the PRNT are comparable; however, the BioPlex MMRV assay is a quadriplex assay, which is advantageous if looking at multiple markers, but it may not be cost-effective if only one target is being investigated.

This study has some limitations. There was no information about the vaccination status of the individuals from which the serum samples were drawn, and therefore, it is not known whether the antibodies were produced by vaccination or natural infection. While we presume that the panels tested are representative of the general population and include a significant proportion of vaccinated individuals, the relative sensitivity for detecting vaccine-induced antibodies could not be determined. It will be important for futures studies to correlate the sensitivity of detection to vaccination status. The VaccZyme gpEIA is quantitative only up to 810 mIU/ml, and therefore, correlation with the BioPlex 2200 above this titer could not be determined, although all the high-titer specimens were positive with both BioPlex 2200 and VaccZyme gpEIA. Furthermore, while the VaccZyme gpEIA measures functional glycoprotein antibody levels, the BioPlex assay only measures total antibody levels and does not assess the antibody affinity or response.

In conclusion, we have shown that the BioPlex 2200 VZV IgG assay can be adapted as a quantitative test using a calibration curve and appropriate cutoffs. The relative specificity was the same as that of the VaccZyme gpEIA reference test, and the relative sensitivity was 97.4%. This performance makes the BioPlex 2200 suitable for high-throughput use in seroepidemiology studies.
